# Endovascular Treatment with Drug-Eluting Balloon for Severe Subclavian Artery Stenosis Involving the Origin of the Vertebral Artery

**Published:** 2020-02-20

**Authors:** E Dinoto, F Pecoraro, D Mirabella, F Ferlito, A Farina, N Lo Biundo, P Conti, G Bajardi

**Affiliations:** Vascular Surgery Unit; Department of Surgical, Oncological and Oral Sciences, University of Palermo

**Keywords:** subclavian artery stenosis, balloon angioplasty, steal syndrome, drug eluting balloon

## Abstract

The first line approach for subclavian steal syndrome is PTA-stenting of subclavian artery. When the ipsilateral vertebral artery origin is involved or in closed proximity of the atherosclerotic lesion in the subclavian artery PTA-stenting is at risk of ipsilateral vertebral artery coverage. Herein we report our experience with DEB to address lesions involving the subclavian artery and the origin of the ipsilateral vertebral artery. From January 2017 to February 2019, patients presenting subclavian artery lesion involving the origin of the ipsilateral vertebral artery and treated using primary DEB, were included. Three patients, with left subclavian steal syndrome, were identified. The perioperative mortality and morbidity were outcomes evaluated. Freedom from occlusion, secondary patency, amputation rate was registered. A total of 3 (2 female) patients were included in the study. No complication, symptoms recurrence, restenosis or occlusion were reported at duplex scan during 12-month follow-up. Indication for stenting was arterial dissection. In our limited experience, the use of DEB in association to embolic protection device in the treatment of atherosclerotic subclavian lesion involving the origin of the vertebral artery was safe and technically feasible.

## I. INTRODUCTION

The first line approach of subclavian steal syndrome is PTA-stenting of subclavian artery. When the ipsilateral vertebral artery origin is involved or close to the atherosclerotic lesion in the subclavian artery, PTA-stenting is at risk of ipsilateral vertebral artery coverage: in this case, the open surgical treatment is indicated. However, applying the knowledge gained by use of Drug eluting balloon (DEB) in peripheral artery disease, we think it’s possible to extend the endovascular treatment also in vertebral artery involving cases. Herein we report our experience with DEB to treat lesions involving the subclavian artery and the origin of the ipsilateral vertebral artery.

## II. METHODS

**Figure 1:**
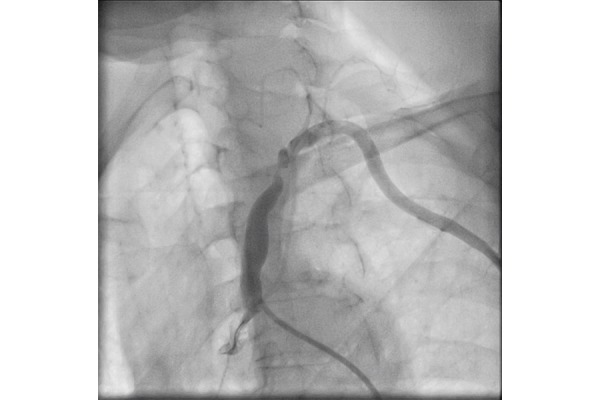
Left subclavian artery stenosis, in patient with subclavian steal syndrome.

**Figure 2:**
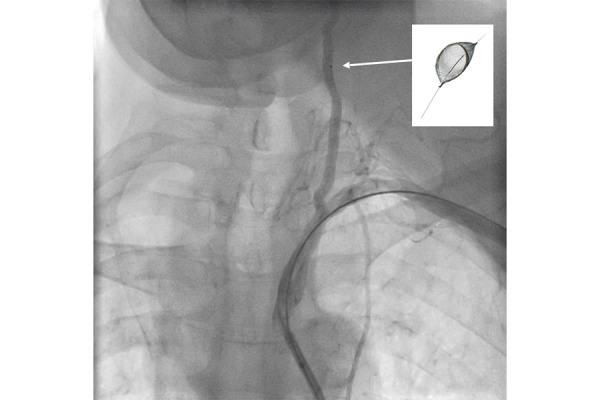
Embolic protection device deployed inside the ipsilateral vertebral artery.

A retrospective analysis of patients with subclavian steal syndrome, addressed with endovascular procedures, was conducted. From January 2017 to February 2019, were included patients presenting subclavian artery lesion involving the origin of the ipsilateral vertebral artery and treated using primary DEB (Fig. 1). Three patients with left subclavian steal syndrome, were identified. All cases were treated with a percutaneous transfemoral approach, using an embolic protection device located in ipsilateral vertebral artery (Fig. 2). The outcomes evaluated were perioperative mortality and morbidity. Freedom from occlusion, secondary patency, amputation rate was registered. Addition maneuvers including stenting or angioplasty with drug eluting balloon (DEB) were reported.

## III. RESULTS

**Figure 3:**
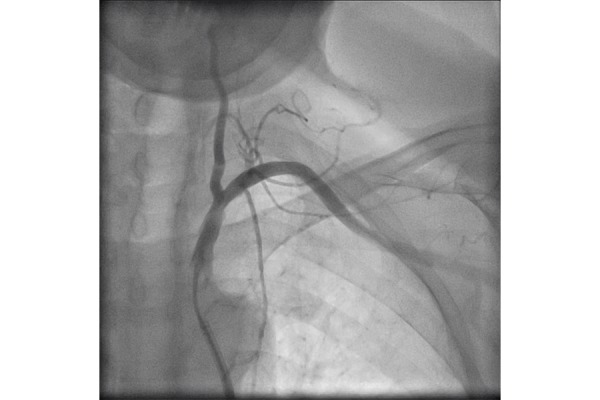
Left vertebral and subclavian arteries after DEB procedure.

A total of 3 (2 female) patients with a mean age of 66 years were included in the study. Technical success was achieved in all cases (Fig. 3). After the angioplasty using DEB, an additional stenting procedure was required in one case because of a proximal dissection: the origin of ipsilateral vertebral artery was not covered during stent released. At end of procedure, all patients recovered from the preoperative clinical condition. No complication, symptoms recurrence, restenosis or occlusion were reported at duplex scan during 12-month follow-up. Indication for stenting was arterial dissection.

## IV. DISCUSSION

Traditional open surgical revascularization treatments have shown excellent long-term durability for supra-aortic vessels. Neurological manifestations such as dizziness, visual disturbances, and stroke indicate failure perfusion of the central nervous system, which is a significant predictor of mortality^1^. However, the risk of stroke and TIA recurrence in symptomatic patients may be three times higher compared with patients without stenosis^2^. Endovascular treatment is the first therapeutic option for subclavian artery occlusive lesions, and generally provides positive results^3^. In literature, there are few cases of endovascular treatment in still syndrome vertebral artery involving. Maciejewski et al. reports an experience of simultaneous vertebral and subclavian artery stenting with good outcome in terms of patency and procedure success rates using DEB for cases of intrastent restenosis^4^. The experience with DEB in peripheral artery disease has showed a reduction of the number of stents and often of their length after PTA^5^. Thus, we think that DEB can be an efficient and safe treatment option for subclavian steal syndrome.

## V. CONCLUSIONS

In our limited experience, the use of DEB in association to embolic protection device in the treatment of atherosclerotic subclavian lesion involving the origin of the vertebral artery was safe and technically feasible. The use of DEB represents an additional tool for patients with complex subclavian lesions involving the vertebral artery origin that are unfit for conventional surgery or when standard PTA stenting is at high risk of vertebral artery coverage.
